# Posterolateral inter-transverse lumbar fusion in a mouse model

**DOI:** 10.1186/1749-799X-8-2

**Published:** 2013-01-23

**Authors:** Justin Bobyn, Anton Rasch, David G Little, Aaron Schindeler

**Affiliations:** 1 Orthopaedic Research & Biotechnology Unit, The Children’s Hospital at Westmead, Locked Bag 4001, Westmead, NSW, 2145, Australia; 2 Discipline of Paediatrics and Child Health, Faculty of Medicine, University of Sydney, Sydney, Australia

**Keywords:** Spine, Fusion, Arthrodesis, Mouse, BMP

## Abstract

**Background:**

Spinal fusion is a common orthopaedic procedure that has been previously modeled using canine, lapine, and rodent subjects. Despite the increasing availability of genetically modified mouse strains, murine models have only been infrequently described.

**Purpose:**

To present an efficient and minimally traumatic procedure for achieving spinal fusion in a mouse model and determine the optimal rhBMP-2 dose to achieve sufficient fusion mass.

**Method:**

MicroCT reconstructions of the unfused mouse spine and human spine were compared to design a surgical approach. In phase 1, posterolateral lumbar spine fusion in the mouse was evaluated using 18 animals allocated to three experimental groups. Group 1 received decortication only (n = 3), Group 2 received 10 μg rhBMP-2 in a collagen sponge bilaterally (n = 6), and Group 3 received 10 μg rhBMP-2 + decortication (n = 9). The surgical technique was assessed for intra-operative safety, efficacy, access and reproducibility. Spines were harvested for analysis at 3 weeks (Groups 1, 2) and 1, 2, and 3 weeks (Group 3). In phase 2, a dose response study was carried out in an additional 18 animals with C57BL6 mice receiving sponges containing 0, 0.5, 1, 2.5, 5 μg of rhBMP-2 per sponge bilaterally.

**Results:**

The operative procedure via midline access was rapid and reproducible, and fusion of the murine articular processes was found to be analogous to the human procedure. Unlike reports from other species, decortication alone (Group 1) yielded no new bone formation. Addition of rhBMP-2 (Groups 2 and 3) yielded a significant bone mass that bridged the L4-L6 vertebrae. The subsequent dose response experiment revealed that 0.5 μg rhBMP-2 per sponge was sufficient to create a fusion mass.

**Conclusion:**

We describe a new approach for mouse lumbar spine fusion that is safe, efficient, and highly reproducible. The technique we employed is analogous to the human midline procedure and may be highly suitable for genetically modified mouse models.

## Introduction

Spinal fusion surgery has been performed since the pioneering work of Hibbs [1], Albee [2] and DeQuervain in the early 20th century. When successful the procedure has significantly reduced the morbidity associated with spinal deformity and, controversially, discomfort. Historically, the procedure has suffered from high rates of non-union. It has been estimated that 5-30% of spinal fusion procedures result in pseudarthrosis [3], the causes of which are certainly multifactorial.

Animal surgical models have been utilized extensively to improve surgical technique and gain insight into the mechanisms of spinal fusion failure. Larger animals such as canine [4-8] and ovine subjects [9,10] have traditionally been employed due to their close approximation of the size and anatomy of human patients. Rat and rabbit studies [11-21] typically represent the lower limit with respect to size that spine fusion procedures have been attempted. Mouse models have rarely been studied, likely owing to the technical difficulty associated with surgical access and technique demanded by their small size. Until recently, spinal fusion attempts in mice have been limited to percutaneous injection of BMP solution and mesenchymal stem cells into the paraspinal musculature [22,23], thereby obviating the need to surgically expose and manipulate the vertebral structures.

Advancements in transgenic mice have encouraged the use of surgical models that explore the impact of genetics on repair and regeneration. In 2007, Rao et al. ([24]) proposed a technique for performing posterolateral lumbar spinal fusion in mice, using a modified version of the paraspinal approach described by Wiltse [25]. Fusion was induced via autogenous bone graft from donor mouse of same strain or rhBMP-2 delivered in a collagen sponge. Radiographic assessment demonstrated that a dense fusion mass was formed in 62% of fusion sites, although no specific breakdown was given for the bone graft and rhBMP-2 groups. This model also featured a prolonged operating time of 60 min.

In this study we have utilized a surgical approach involving a single midline incision through the thoracolumbar fascia that more closely approximates the current human midline surgical technique. Such an approach aimed to minimize tissue trauma as well as increase visibility and access to the articular processes. One other aim of this modified procedure was to minimize the operative time and maximize the experimental reproducibility between subjects and the rate of spine fusion.

## Methods

### Anatomical Analysis by MicroCT

The spines of 5 C57BL6 mice, aged between 8 and 10 weeks, were harvested and analyzed via microCT using a SkyScan 1174 compact microCT scanner (SkyScan, Kontich, Belgium). Samples were scanned in 70% ethanol at 21.3 um magnification, 0.5 mm aluminium filter, 50 kV X-ray tube voltage and 800 μA tube electric current. The images were reconstructed using NRecon, version 1.5.1.5 (SkyScan). A global threshold was used representing bone tissue. Representative three-dimensional fractures were reconstructed with sagittal slices using CTVol Realistic Visualisation software version 2.1.0.0 (SkyScan). The structure of the lumbar spine was documented and compared with the relevant human anatomy. Subsequently, 5 additional mice were culled and the lumbar spine was dissected. The relationships of the soft tissue to the bone were observed and documented with a view to establishing a clear and efficient surgical access to the lumbar vertebrae which would allow secure positioning of a collagen sponge for the delivery of osteoblast stimulating factors.

### Surgical model

18 C57BL6 mice (male and female aged 8–10 weeks) received posterolateral lumbar spine fusion surgery. Surgery was performed by a two surgeons in a single sitting with anesthetic support from an experienced animal technician. Group 1 received decortication only (n = 3), Group 2 received 10μg rhBMP-2 in a collagen sponge bilaterally (n = 6), and Group 3 received 10 μg rhBMP-2 + decortication (n = 9).

Mice were anaesthetized with ketamine (35 mg/kg)/xylazine (5 mg/kg) solution administered intraperitoneally. Hair overlying the operative site was shaved with an electric razor once anaesthetized and the area prepped with iodine. Animals were positioned prone with folded gauze beneath the abdomen, increasing the excursion of the lumbar spine to facilitate access to and visibility of the surgical field. The head was covered with saline soaked gauze to protect the ears from overheating under operating lights. rhBMP-2 marketed under the brand name Infuse^®^ (Medronic Australasia) was used in this study. Prior to surgery, the collagen sponge that was supplied with the vial of BMP for human use was cut into 2 mm × 7 mm sections. A solution of saline and BMP was made allowing the accurate pipetting of 10 μg of BMP onto each sponge intra-operatively.

**Figure 1:**
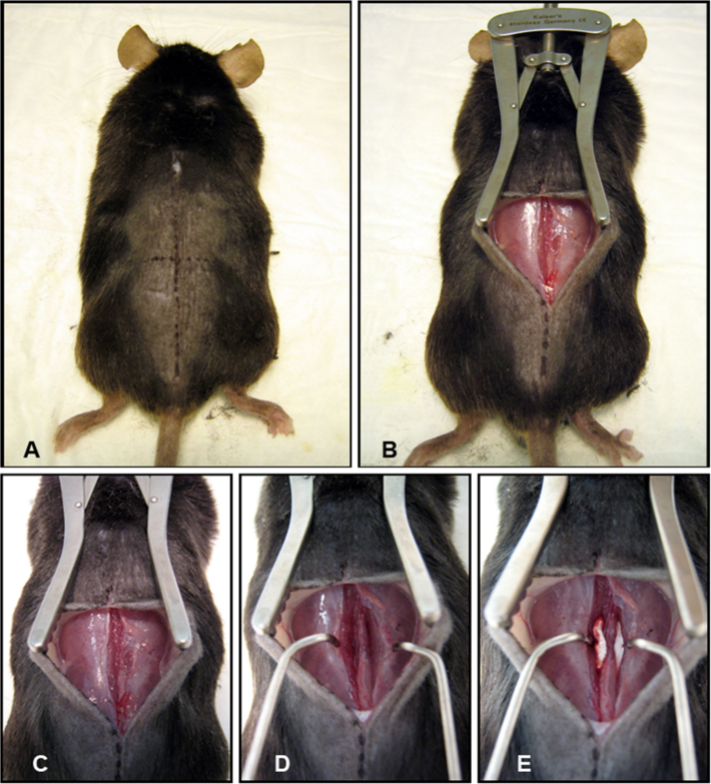
**Following the administration of anesthetic the mouse was positioned prone.** A marker was used to mark the location of the spinous processes, vertical line, and pelvic crest, horizontal line (**A**). A line drawn across the pelvic crests corresponds to the position of the L5-L6 interspace. (**B**). An incision was made through the skin which was the held by a self-retaining retractor. **(C)** A single incision was made through the thoracolumbar fascia over the spinous processes and the paravertebral musculature was dissected by scraping a blade down along the spinous processes and pulling laterally. The dissection of the local musculature forms a pocket on either side of the spine, bounded medially by the spinous processes and inferiorly by the articular processes (**D**). The articular processes are consequently exposed and visualized using a small muscle retractor of our own design. Articular processes are subsequently decorticated with a 1 mm round diamond burr until punctate bleeding is observed. Following decortication of the visualized articular processes, a 7 mm × 2 mm collagen sponge impregnated with either saline or saline/rhBMP-2 solution is inserted into each of the pockets in contact with the posterior elements of the spine (**E**). The muscle and skin are then closed with continuous sutures using 5.0 Vicryl^®^.

With the intention of fusing the lumbar spine from L4-L6, surface anatomy was located to determine incision point. The iliac crest is approximately level with the L5-L6 interspace [24]. This level as well as the midline was marked with surgical pen (Figure 1A). The procedure was performed under an operating microscope or surgical loupes at 2.5× magnification. A 15 mm incision was made in the skin made along the midline, centered over the line running between the iliac crests (Figure 1B). The skin was opened and held in place with a self-retaining retractor (Figure 1C). At this point, the midline was identified by the spinous processes. The iliac crest was directly visible. A second 15 mm incision, through the dorsolumbar fascia, was made in the midline, along the spinous processes. The spinous processes and lumbar paravertebral muscles have now been accessed. The paravertebral muscles overlying the articular processes of L4-L6 were separated from the spinous processes in a single motion by scraping a # 10 blade down the lateral border of the spinous process and pulling the muscles laterally (Figure 1D). This motion shears the muscle at its junction to the bone minimizing trauma to the tissue. This was performed bilaterally exposing the articular processes and creating a potential space between muscle and bone. Muscle was retracted bilaterally with a purpose-built micro-retractor allowing controlled and gentle retraction while maintaining maximum visibility of all articular processes. A pneumatic 1 mm round tip diamond burr was used to decorticate the visible articular processes until punctuate bleeding was observed. Care was taken not to disturb surrounding muscle tissue.

Two collagen sponges, impregnated with either saline or an rhBMP-2/saline solution (containing 10 μg of BMP per sponge), were placed bilaterally in the potential space which has been created (Figure 1E). The fascia was apposed and closed with a single line of continuous sutures using 6.0 Vicryl, The skin was subsequently closed in the same fashion. Mice were placed on a heat pad following surgery and monitored for recovery. Antibiotics were administered in the drinking water for 24 hours postoperatively.

### Study design

Once the safety and efficacy of the surgical model had been demonstrated, it was applied to a small study involving 15 C57BL6 mice to determine the optimal dose of rhBMP-2 required to establish fusion of the spine. Mice were divided into dose groups of 0, 0.5, 1, 2.5, and 5 μg of rhBMP-2 per sponge bilaterally and culled at 3 weeks. Spines were harvested and fixed in 4% paraformaldehyde for 24 hours and then analyzed by X-ray and microCT were used to visualize new bone formation.

### Radiography

Following harvesting and fixation in 4% paraformaldehyde, microCT data was obtained via SkyScan 1174 compact microCT scanner (SkyScan, Kontich, Belgium). Samples were scanned in 70% ethanol at 21.3 um magnification, 0.5 mm aluminum filter, 50 kV X-ray tube voltage and 800 μA tube electric current. The images were reconstructed using NRecon, version 1.5.1.5 (SkyScan). A global threshold was used representing bone tissue. Representative three-dimensional fractures were reconstructed with sagittal slices using CTVol Realistic Visualization software version 2.1.0.0 (SkyScan). For the purposes of bone volume and density calculation, the bone of the fusion mass was isolated from native bone by means of manually drawn regions of interest (ROI).

### Histology

Harvested vertebrae and surrounding musculature were fixed in 4% paraformaldehyde for 24 hours at 4°C on a shaker were and then stored in 70% ethanol until ready for decalcification. Samples were decalcified in 0.34M EDTA (pH 8.0) solution at 4°C on a shaker for 30 days with solution changes every 2–3 days. Samples were next embedded in paraffin and sectioned transversely through the center of the fusion mass at a thickness of 5 microns. Mounted sections were stained via Picro Sirius Red/Alcian Blue to differentiate bone and cartilage.

### Statistics

The significance of the medians and distribution of bone volume data as a function of increasing dose was first analyzed via stringent non-parametric statistics (Kruskal-Wallis independent sample test; SPSS Statistics v18). Using an assumption of normally distributed data, an ANOVA with *post-hoc* Bonferroni test was then performed to compare individual groups. The limit for statistical significance was set at 0.05. Correlation was tested using a Pearson product–moment correlation test (Excel 2010) to generate the R-value. Significance was tested by calculating the t-value using the formula t = (r*(n-2)^0.5)/((1-r^2)^0.5) and significance calculated using the *tdist* function.

### Ethics

All animal experimentation was performed with ethics approval granted by the Westmead Hospital Animal Ethics Committee under protocol 5056.

## Results

### Anatomical examination of the unfused mouse spine

**Figure 2:**
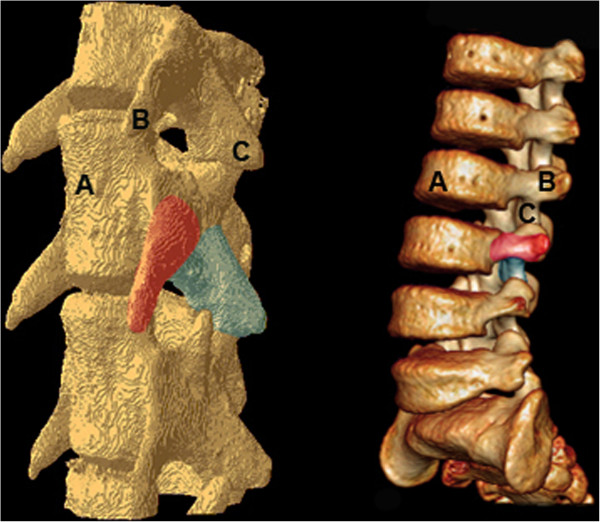
**Three-dimensional reconstructions of CT images demonstrating the anatomy of murine lumbar spine (left) vs human lumbar spine (right).** (**A**) Anterior vertebral body. (**B**) Transverse process. (**C**) Inferior articular process. The position and orientation of the transverse process of the mouse spine differs significantly from the human spine. Both the murine inferior articular process and the human transverse process share a lateral orientation.

MicroCT images of the mouse lumbar spine were compared to human anatomical scans (Figure 2). Comparison of both species showed shared similar vertebral structures, although their position and orientation differed between species. Of particular note was the positioning of the transverse process (TP) in the mouse lumbar vertebrae. In the human, the TP is located posterolaterally, and is therefore the anchor point in posterolateral spine fusion. In contrast, in the mouse it is the inferior articular processes, rather than the transverse processes that share this orientation. For the purposes of modeling a human surgery in a mouse, we concluded that a posterolateral interarticular process spine fusion would more closely approximate the posterolateral intertransverse procedure used in humans today.

### Surgical timing and morbidity

There was no intra- or post- operative morbidity or mortality in either the fusion outcome study or the rhBMP-2 dose response study. Animals were monitored for neurological impairment (particularly of the hind limbs), wound infection, weight loss and distressed behavior. Operative time, in the hands of an experienced surgeon, was approximately 15 minutes skin to skin. We believe that the brief operating, and hence anesthetic time as well as the minimized trauma contributed significantly to the observed safety of the procedure.

### Fusion outcomes

**Figure 3:**
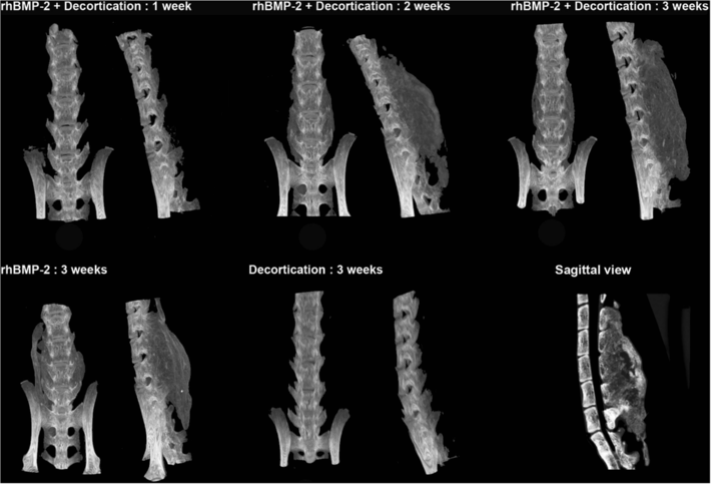
**Volume of the BMP induced fusion mass was assessed via microCT using a Skyscan 1174 scanner.** A fusion mass was only produced in groups in which BMP was implanted. No new bone was observed at 1 week post op, while maximum fusion volume was achieved by week 2 and maintained at week 3. Decortication of the articular processes alone was insufficient to achieve new bone formation. A reconstructed sagittal view of a fused spine at 3 weeks is shown at the lower right. The section is taken from the midline, a dense fusion mass uniting the vertebrae of L1-L5.

**Figure 4:**
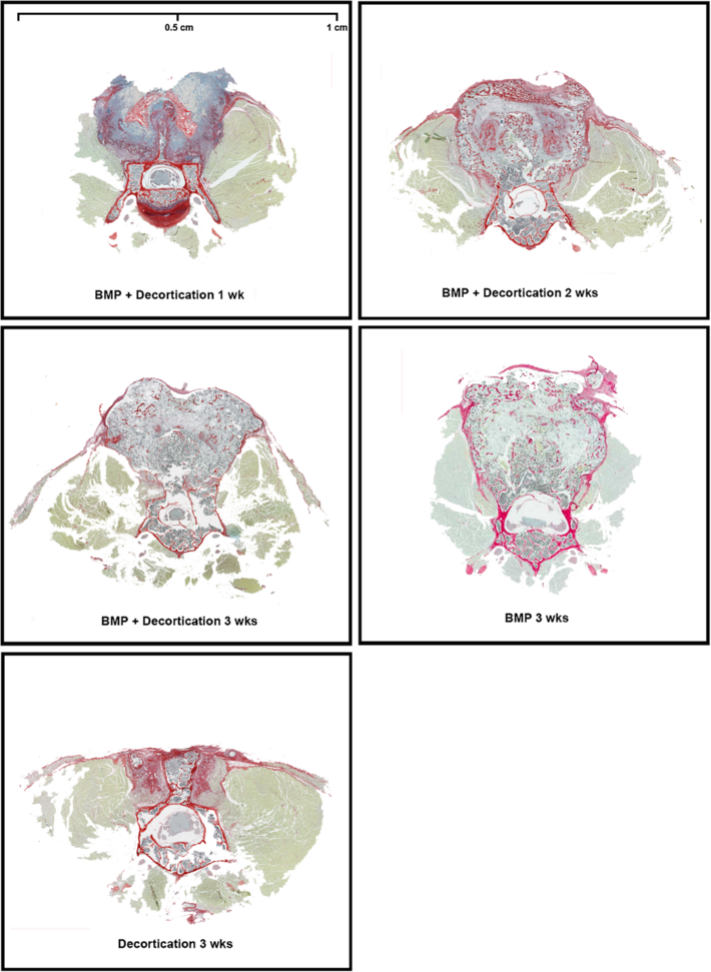
**Histological samples retrieved from fused murine vertebrae.** Samples have been stained with Pico Sirius red and Alcian blue stain to differentiate between bone and collagen. At 1 week, a large collagen mass overlaying the posterior elements of the vertebrae forms with disruption of local musculature. By the second week postoperatively, a large bony mass that was continuous with the cortex of the vertebrae was present. This mass exhibited a resorptive marrow-like center by week 3. No new collagen or bone was observed in the decortications only spine.

Our results were consistent with previous studies [24], demonstrating little or no bone growth evident in decortication-only control animals on micro-CT reconstruction as well as in decortication/BMP animals culled at 1 week. By week 2, a significant volume of bone was observed in all animals which received BMP-2. The bone bridge typically united all vertebrae from L2/3-L6 with an AP dimension equal to or greater than that of the lumbar vertebrae to which it has fused (Figure 3). The bone mass was homogenously of lower density that the vertebral cortical bone. At week 3, areas of centralized resorption were noted in the bone mass. Decortication does not appear to have contributed significantly to new bone formation. MicroCT reconstructions were supported by histology (Figure 4). Paraffin sections stained with Picro Sirius Red/Alcian Blue stain demonstrated an abundance of collagen, stained blue, overlying the posterior elements of the spine. The observed collagen is likely a remnant of the collagen sponge used to deliver the rhBMP-2 and was associated with lateral displacement of the perispinal musculature, stained green. No new bone formation, stained red, was observed at 1 week time points in any group. Overwhelming bone formation was observed at the 2 week end point in all groups dosed with rhBMP-2. The size of the fusion mass was unchanged at 3 weeks. However, consistent with microCT data, an area of centralized resorption was evident. This was visualized as a reduction of red staining structures in the center of the fusion mass relative to samples obtained at 2 weeks.

### rhBMP-2 dose response study

**Figure 5:**
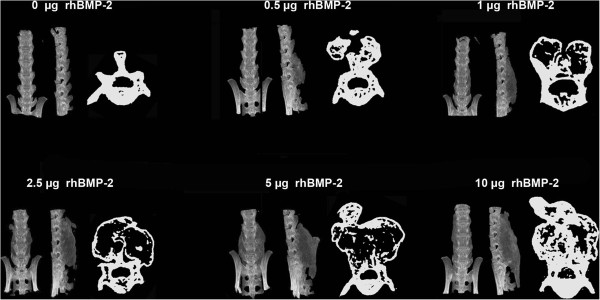
**AP and lateral microCT images demonstrating the volume of new bone formed in response to rhBMP-2 dose.** To the right of each set of microCT images was a cross sectional image of the spine taken at its widest point in the transverse plane. An increase in the size of the fusion mass was observed with increasing rhBMP-2 dose. It was noted that at a dose of 0.5 μg sufficient bone was formed to bridge the L4-L6 vertebrae resulting in successful fusion.

**Figure 6:**
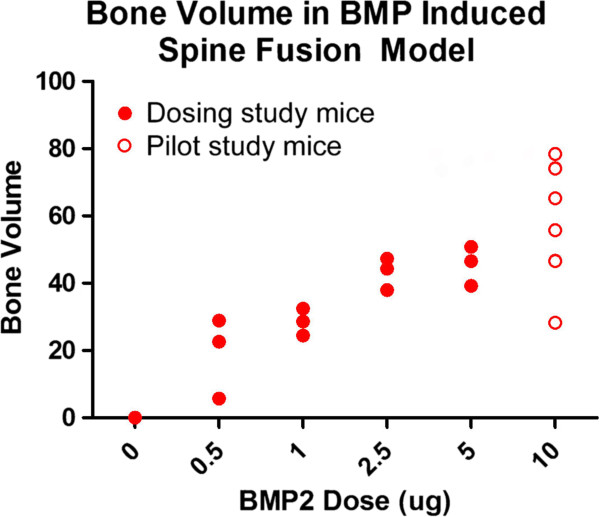
**Volume of spine fusion bone mass as a function of rhBMP-2 dose.** A dose–response relationship was observed with consistent bone mass being formed with a dose as low as 0.5 μg of rhBMP-2. Bone volume data for the 10 μg dose (n = 6) is included from the first phase and was consistent with the dose–response trend.

A dose responsive relationship was observed between the rhBMP-2 dose and size of the fusion mass (Figure 5). When collagen sponges were added with 0μg of rhBMP-2, no fusion mass was observed. MicroCT data showed that a dose as low as 0.5 μg was able to promote fusion and the amount of bone formed increased with increasing rhBMP-2 dose (Figure 6). The significance of the distribution of bone volume data as a function of increasing rhBMP-2 dose was found to be different by non-parametric independent sample analysis (P < 0.01) and also by ANOVA (P < 0.01). *Post-hoc* intergroup testing showed significant differences between the induced bone volumes between the 0 μg (no treatment) group and the 2.5 μg, 5 μg, and 10 μg rhBMP-2 groups (all P < 0.01). Bonferroni *post-hoc* intergroup testing showed significant differences between the induced bone volumes between the 10 μg groups and the 0 μg (0.000), 0.5 μg (0.005) and 1 μg (0.051) groups as well as between the 0 μg and the 2.5 μg (0.008) and 5 μg (0.005) groups. Based on the increasing dose responsiveness, the correlation between rhBMP-2 and bone volume formed was tested using a Pearson correlation test and confirmed a strong linear relationship (R = 0.74, P < 0.01).

## Discussion

Historically, larger animals have been used to model spine fusion surgery due to their proximity in size and anatomy to humans. Recent advancements in transgenic mice have generated an experimental niche for the use of murine surgical models. Knockout and transgenic mice offer a unique opportunity for insight into the genetic and cellular mechanisms underlying bone repair [26]. Idiopathic scoliosis (IS) is the most common cause of spinal deformity in children, but the etiology of the disease has remained elusive. Recent research has found a strong association between mutations of the CHD7 gene and the development of IS [27]. The potential exists to replicate CHD7-mutation dependent IS in transgenic mice and to investigate intervention therapies in an accurate murine model. Similar advantages are offered to the modeling of scoliosis in neurofibromatosis type 1 (NF1) patients. NF1 is the most common single gene disorder and at least 23% of patients diagnosed with NF1 will show signs of scoliosis in childhood [28,29]. A mouse model for modeling human NF1 deficiency is currently available, and while it does not spontaneously develop scoliosis it shows impaired long bone healing and response to rhBMP treatment [30,31]. There are several additional advantages using the mouse as an experimental organism. The small size of the mouse, though technically challenging, facilitates relatively quick operations and allows for low intra-operative morbidity and mortality. Furthermore, when combined with its short generation time and inexpensive housing costs, makes it a valuable high throughput model.

A major drawback to using the mouse to replicate human surgery lies in the anatomical differences between the two species. With respect to the lumbar spine, our investigation into the murine anatomy yielded several distinct morphological differences between the mouse and the human lumbar vertebrae which have been previously overlooked, particularly the position and orientation of the transverse processes. However, the structural similarities of the mouse articular processes and the human transverse processes may be exploited to yield a procedure that is functionally analogous to that which is performed in humans.

Previous mouse and rat models of posterolateral spine fusion have adopted a modification of the Wiltse technique which utilizes a paraspinal approach to the transverse processes [19,24,25]. Given the anatomical differences between rodents and humans we don’t believe that this is an optimal approach. Furthermore, the Wiltse technique uses twice as many incisions as a midline approach and is not currently in popular practice. The result is a procedure that does not mimic contemporary technique or relevant anatomy and leads to longer operating times with a consequent risk of increased morbidity and mortality. The surgical approach used in this study minimizes the number of incisions, tissue trauma and ultimately the total operative time. In mice who only received articular process decortication as intervention, no bone formation was observed. Additionally, little difference was observed by the addition of decortication to the mice receiving rhBMP-2 suggesting intrinsic intraspecies differences with larger animals. Canines have been reported to readily fuse following decortication alone [32], and decortication is also a common clinical practice in spine fusion surgery.

Our initial study used an rhBMP-2 dose of 10μg per sponge, based upon scaling down doses commonly used to create a large bone mass in rabbits [33,34]. This yielded a very large amount of bone formation which is equal to or greater in volume than the spine itself. Such a large mass of bone suggests that the mouse spine is particularly sensitive to the osteogenic effects of rhBMP-2 and that a functional and stabilizing fusion mass could be achieved with a lower dose. We propose that the overwhelming response to the 10 μg dose may be obscuring the influence of additional variables, such as decortications of the articular processes. A follow-up study was performed to determine the minimum dose of BMP-2 required for fusion. A dose response relationship between rhBMP-2 and the volume of the 3 week fusion mass was observed. It was found that a dose of 0.5 μg, equivalent to 5% of the original dose, was sufficient to achieve successful fusion. The resulting fusion mass was significantly smaller than that achieved using 5 μg or 10 μg rhBMP-2. This smaller fusion mass may be more sensitive to the effects of intervention.

In summary, we have demonstrated that a midline approach to posterolateral interarticular process spine fusion in a mouse model is safe, efficient, and yields consistent and reproducible results. A BMP-2 dose of 0.5 μg per sponge was sufficient to achieve fusion of adjacent vertebrae. By exploiting anatomical similarities between the species, the technique closely mirrors that which is used in humans. The development of a surgical protocol in mice that is functionally analogous to the procedure in human patients is advantageous. This model permits the rapid observation of a large number of subjects over a relatively short period of time and allows valid extrapolation of reproducible data and results, thereby fulfilling the criteria for the ideal animal model as proposed by Schimandle and Boden [20].

## Competing interests

The authors declare that they have no competing interests.

## Authors’ contributions

DGL, AS, JB and AR for study design and conceptualization. JB, AR, LP and MK for study conduct. JB for data analysis. DGL, AS, JB for data interpretation. JB for drafting manuscript. DGL and AS for manuscript revision and approval. All authors read and approve the final manuscript.
